# Usefulness of Palliative Prognostic Index, Objective Prognostic Score, and Neutrophil–Lymphocyte Ratio/Albumin Ratio As Prognostic Indicators for Patients Without Cancer Receiving Home-Visit Palliative Care: A Pilot Study at a Community General Hospital

**DOI:** 10.1089/pmr.2023.0096

**Published:** 2024-04-04

**Authors:** Taiki Hori, Ken-ichi Aihara, Koki Ishida, Kaori Inaba, Keisuke Inaba, Yousuke Kaneko, Keisuke Kawahito, Shoki Bekku, Minae Hosoki, Kensuke Mori, Kanako Itami, Masayo Katsuse, Yoshimi Hanaoka, Teruyoshi Kageji, Hideyuki Uraoka, Shingen Nakamura

**Affiliations:** ^1^ Department of Internal Medicine, Tokushima Prefectural Kaifu Hospital, Tokushima, Japan.; ^2^ Department of Hematology, Endocrinology and Metabolism, Tokushima University Graduate School of Biomedical Sciences, Tokushima, Japan.; ^3^ Department of Community Medicine and Medical Science, and Tokushima University Graduate School of Biomedical Sciences, Tokushima, Japan.; ^4^ Department of General Medicine, Tokushima University Graduate School of Biomedical Sciences, Tokushima, Japan.; ^5^ Department of Nursing, Tokushima Prefectural Kaifu Hospital, Tokushima, Japan.; ^6^ Department of Neurosurgery, and Tokushima Prefectural Kaifu Hospital, Tokushima, Japan.; ^7^ Department of Orthopedic Surgery, Tokushima Prefectural Kaifu Hospital, Tokushima, Japan.

**Keywords:** chronic obstructive pulmonary disease, disease progression, heart failure, home-visit palliative care, prognostication, serious illness

## Abstract

**Background:**

Although the palliative prognostic index (PPI), objective prognostic score (OPS), and neutrophil–lymphocyte ratio/albumin ratio (NLR/Alb) are well-known prognostic indicators for cancer patients, they do not provide clarity when it comes to predicting prognosis in patients without cancer who receive home-visit palliative care.

**Objective:**

The aim of this study was to determine whether PPI, OPS, and NLR/Alb can predict prognosis for patients without cancer who received home-visit palliative care.

**Design:**

This is a retrospective study.

**Setting/Subjects:**

We recruited 58 patients without cancer who received home-visit palliative care from Tokushima Prefectural Kaifu Hospital, Japan, and died at home or at the hospital within seven days of admission between January 2009 and March 2023.

**Measurements:**

The PPI, OPS, and NLR/Alb of the study patients were evaluated at regular intervals, and statistical analysis was performed on the relationship between these indices and the time to death.

**Results:**

Simple regression analysis showed that PPI, OPS, and NLR/Alb were negatively correlated with the period until death (*p* < 0.001). The survival curves of the groups classified according to PPI, OPS, and NLR/Alb were significantly stratified. The predictive capacities of PPI, OPS, and NLR/Alb for death within 21 days were as follows: PPI (area under the curve [AUC]: 0.71; sensitivity: 59%; specificity: 68%), OPS (AUC: 0.73; sensitivity: 88%; specificity: 47%), and NLR/Alb (AUC: 0.72; sensitivity: 72%; specificity: 73%).

**Conclusions:**

PPI, OPS, and NLR/Alb were useful in predicting the survival period and short-term prognosis within 21 days for patients without cancer who received home-visit palliative care.

## Introduction

Prognostic prediction is difficult for patients without cancer.^1^ Disease progression can be divided into three patterns depending on type, as described in illness trajectories.^2,3^ As cancer patients reach the end of their lives quickly due to relatively rapid deterioration in the last few months of care, their prognosis prediction is well established. Patients with acutely exacerbating illness as represented by noncancerous conditions, such as chronic respiratory disease and heart failure, are repeatedly hospitalized. Death occurs as organ failure progresses. Geriatric syndromes and dementia tend to progress slowly and often change slowly, even in their final stages. Prognosis prediction is crucial for determining the treatment methods and location of recuperation, but there were fewer prognostic indicators for patients without cancer.

For cancer patients, decision-making support at the end of life is provided based on prognosis prediction, such as the palliative prognosis (PaP) score,^4^ palliative prognostic index (PPI),^5^ objective prognostic score (OPS),^6^ and the Prognosis in Palliative Care Study predictor model.^7^ Zhao et al. reported the neutrophil–lymphocyte ratio/albumin ratio (NLR/Alb) as a novel prognostic index for predicting postoperative survival of patients with esophageal squamous cell carcinoma.^8^ The importance of palliative care for patients without cancer has also been indicated in recent years, but there is a lack of research regarding patients without cancer and decision-making support based on prognosis prediction for them.

The world's population is aging rapidly. Japan has the highest proportion of older adults worldwide.^9^ Considering limited medical resources, discussion of patient management, services needed, and financial implications is required. Hospice is reserved for patients with cancer and acquired immunodeficiency syndrome (AIDS) according to current insurance regulations in Japan. Patients with other diseases spend their time in long-term and palliative care beds, nursing homes, or at home. In total, 54.6% of Japanese citizens aged 55 years or more hoped to die at home,^10^ but the national mortality rate in that setting remains at 10–20%.

Japan has the most prolonged hospitalization in Organization for Economic Cooperation and Development (OECD) member countries.^11^ Moreover, the Japanese government promotes home care. However, regional disparities exist in home care due to the uneven distribution of doctors and medical institutions.

## Materials and Methods

### Study design, participants, and ethics statement

In this retrospective study, we recruited noncancer patients from Tokushima Prefectural Kaifu Hospital who received home-visit palliative care regardless of the disease. Eligible patients were those who received home-visit care at the discretion of their attending physician and died at home or the hospital within seven days of admission after the start of home-visit care from January 2009 to March 2023. Tokushima Prefectural Kaifu Hospital is a community general hospital in the rural area of Tokushima Prefecture. Kaifu district has ∼18,000 people and only a single emergency hospital.

Therefore, Kaifu Hospital provides a wide range of services, including acute phase treatment, support for home discharge, and home-visit care. Hospitals that provide long-term care or hospice are ∼40 km from ours using the most direct route. The number of nursing homes or satellite hospitals around Kaifu Hospital is limited. Residents and their families living in Kaifu district consider home care an option.

We collected blood test results, excluding hospitalizations, and evaluated PPI and OPS from electronic medical records during the same period. The PPI, OPS, and NLR/Alb of the study patients were evaluated at regular intervals when attending physicians checked blood tests, and statistical analyses were performed to determine the relationship between these indices and time to death.

This study followed the institutional guidelines of Tokushima Prefectural Kaifu Hospital. The study procedures were in accordance with the Declaration of Helsinki, and the study was approved by the relevant institutional review board (approval number 2023009).

### PPI, OPS, and NLR/Alb

PPI was defined using the palliative performance scale (PPS),^12^ oral intake, edema, dyspnea at rest, and delirium (Supplementary Tables S1 and S2). When a PPI of 6.5 or higher was adopted as the cutoff point for terminally ill cancer patients, death within 21 days was predicted with a sensitivity of 80% and a specificity of 85%.^5^ Patients were classified into three groups according to PPI (Group A: 0–2.0, Group B: 2.5–4.0, Group C: 4.5–15.0), and survival curves were analyzed as previously reported for cancer patients.

OPS was defined using the Eastern Cooperative Oncology Group Performance Status (ECOG PS), oral intake, dyspnea at rest, white blood cell count, total bilirubin (Bil), creatinine (Cr), and lactate dehydrogenase levels (Supplementary Table S3). When an OPS of 3.0 or higher was adopted as a cutoff point for terminally ill cancer patients, death within 21 days was predicted with a sensitivity of 74.7% and a specificity of 76.5%.^6^ Patients were classified by OPS into Group A (0–2.0) and Group B (3.0–8.0).

NLR/Alb is the neutrophil-to-lymphocyte ratio (NLR) divided by albumin (g/L) (Alb). Patients with NLR/Alb ≤0.1 displayed significantly better five-year cancer-specific survival than patients with NLR/Alb >0.1 (39.1% vs. 11.0%, *p* < 0.001) among patients with esophageal squamous cell carcinoma.^8^ Patients were classified by NLR/Alb into Group A (NLR/Alb ≤0.1), Group B (0.1 < NLR/Alb ≤0.2), and Group C (0.2 < NLR/Alb).

### Statistical analysis

Continuous variables with a normal distribution are expressed as means ± standard deviation and those with a non-normal distribution are expressed as medians (first quartile [Q1]—third quartile [Q3]). Categorical variables were compared using Fisher's exact test. For comparisons between the two groups, we performed the Mann–Whitney *U* test or Student's *t*-test for numeric variables, depending on the distribution of the variable. We evaluated the relationships between PPI, OPS, NLR/Alb, and time to death using univariate analysis, performed using GraphPad Prism 9 software (GraphPad Software, San Diego, CA).

The median survival time and 95% confidence interval (CI) for each group were calculated using the Kaplan–Meier method, and significant differences between survival curves for each group were evaluated using the log-rank test. The predictive capacities of these indices for death within 21 days were evaluated using the area under the curve (AUC), Hosmer–Lemeshow test, and decision–curve Analysis. The cutoff point for each index was estimated based on the Youden index. These statistical analyses were performed using R (The R Foundation for Statistical Computing, Vienna, Austria), and EZR^13^ (Saitama Medical Center, Jichi Medical University, Saitama, Japan), a graphical user interface for R. Statistical significance was set at *p* < 0.05.

## Results

### Patient characteristics

Patient characteristics are presented in Table 1. We included 58 patients, and 182 evaluations were performed. The average age of the patients was 83 (77–91) years, and the average duration from the start of home-visit palliative care to death was 71 (10–223) days. The proportion of deaths at home was 79.3%, and the other patients died within seven days of hospitalization.

**Table 1.** Patient Characteristics

	Total subjects (***n*** = 58)
Male, *n* (%)	34 (58.6)
Total number of evaluations (times)	182
Number of evaluations per person (times)	2 (1–4)
Median interval of evaluations (days)	37 (12–71)
Period from start of home-visit palliative care to death (days)	71 (10–223)
Data at the start of home-visit palliative care	
Age (years)	83 (77–91)
ECOG PS, *n* (%)	
4	35 (60.3)
1–3	23 (39.7)
Palliative Performance Scale, *n* (%)	
10–20	35 (60.3)
30–50	20 (34.5)
60–100	3 (5.2)
Oral intake, *n* (%)	
Mouthfuls or less	17 (29.3)
Reduced but more than mouthfuls	12 (20.7)
Normal	29 (50.0)
Edema, *n* (%)	20 (34.5)
Dyspnea at rest, *n* (%)	10 (17.2)
Delirium, *n* (%)	4 (6.9)
Underlying disease, *n* (%)	
Dementia	14 (24.1)
Chronic respiratory disease	14 (24.1)
Chronic heart failure	10 (17.2)
Cerebrovascular disease	7 (12.1)
Parkinson's disease	3 (5.2)
Chronic liver disease	3 (5.2)
Chronic kidney disease	3 (5.2)
Others	4 (6.9)
Laboratory data at the start of home-visit palliative care	
WBC ( × 10^3^/μL)	6.03 (5.15–8.42)
Neu ( × 10^3^/μL)	4.48 (3.39–6.22)
Lym ( × 10^3^/μL)	1.14 (0.77–1.60)
Hb (g/dL)	11.4 (9.8–12.8)
Plt ( × 10^3^/μL)	203 (157–253)
Alb (g/L)	27 (23–33)
Total Bil (mg/dL)	0.6 (0.5–0.9)
LDH (U/L)	218 (175–261)
Cr (mg/dL)	0.75 (0.54–1.28)
Prognostic indices at the start of home-visit palliative care	
PPI	5.5 (3.5–6.5)
OPS	2.0 (1.0–2.0)
NLR/Alb	0.17 (0.12–0.33)
Data at time of death	
Age (years)	83 (77–91)
Death at home, *n* (%)	46 (79.3)
Peripheral parenteral nutrition, *n* (%)	16 (27.6)
Total parenteral nutrition, *n* (%)	0 (0.0)
Tube feeding, *n* (%)	8 (13.8)
Oxygen administration, *n* (%)	27 (46.6)
Opioid use, *n* (%)	2 (3.4)

Neu: neutrocytes; Lym: lymphocytes; Hb: hemoglobin; Plt: platelets; Alb: albumin; Bil: bilirubin; Cr: creatinine.

ECOG PS, Eastern Cooperative Oncology Group Performance Status; LDH, lactate dehydrogenase; NLR/Alb, neutrophil–lymphocyte ratio/albumin ratio; OPS, objective prognostic score; PPI, palliative prognostic index; WBC, white blood cell.

### Associations of PPI, OPS, and NLR/Alb with the period until death

**FIG. 1.**
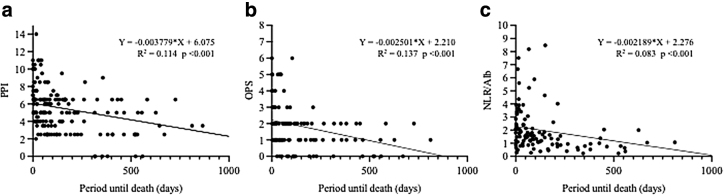
The association between prognostic indices and period until death using univariate analysis for patients without cancer who received home-visit palliative care. **(a)** The association between PPI and survival time. **(b)** The association between OPS and survival time. **(c)** The association between NLR/Alb and survival time. NLR/Alb, neutrophil–lymphocyte ratio/albumin ratio; OPS, objective prognostic score; PPI, palliative prognostic index.

Simple regression analyses showed that PPI, OPS, and NLR/Alb were negatively correlated with the period until death (*Y* = −0.003779X+6.075, R^2^ = 0.114, *p* < 0.001; *Y* = −0.002501X+2.210, R^2^ = 0.137, *p* < 0.001; and *Y* = −0.002189X+2.276, R^2^ = 0.083, *p* < 0.001, respectively) (Fig. 1a–c).

### Comparison of survival curves for each group according to PPI, OPS, and NLR/Alb

**FIG. 2.**
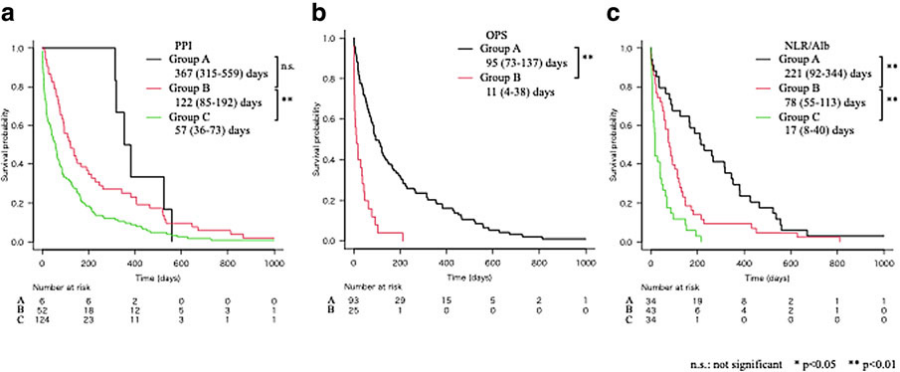
Survival curves of the groups stratified according to PPI, OPS, and NLR/Alb for patients without cancer who received home-visit palliative care. **(a)** Survival curves of the groups classified by PPI (Group A: 0–2.0, Group B: 2.5–4.0, Group C: 4.5–15.0). **(b)** Survival curves of the groups classified by OPS (Group A: 0–2.0, Group B: 3.0–8.0). **(c)** Survival curves of the groups classified by NLR/Alb (Group A: NLR/Alb ≤0.1, Group B: 0.1 < NLR/Alb ≤0.2, Group C: 0.2 < NLR/Alb).

The survival curves of the groups classified according to PPI, OPS, and NLR/Alb are shown in Figure 2. The median survival time and interquartile range of each group classified according to PPI were 367 (315–559), 122 (85–192), and 57 (36–73) days. The survival of each group classified according to OPS was 95 (73–137) and 11 (4–38) days. The survival of each group classified according to NLR/Alb was 221 (92–344) days, 78 (55–113) days, and 17 (8–40) days, respectively. The *p*-value was 0.25 only when comparing the survival curves of Groups A and B classified according to PPI, but was <0.01 when comparing the other survival curves.

### The predictive capacities of PPI, OPS, and NLR/Alb for death within 21 days

**FIG. 3.**
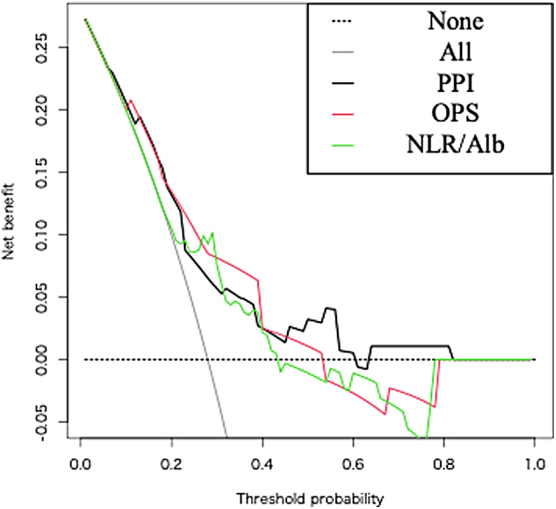
The predictive capacities of PPI, OPS, and NLR/Alb for death within 21 days. The predictive capacities of PPI, OPS, and NLR/Alb were evaluated using decision curve analysis. No significant differences were observed between indices.

Table 2 gives the predictive capacities of PPI, OPS, and NLR/Alb for death within 21 days. The AUC of these indices was 0.70 or higher, indicating high discrimination capacities; however, sensitivity and specificity varied among the indices. The Hosmer–Lemeshow test showed that the predictions for death within 21 days by these indices were well calibrated. The three indices presented no significant differences when evaluated using decision curve analysis (Fig. 3).

**Table 2.** The Predictive Capacities of Palliative Prognostic Index, Objective Prognostic Score, and Neutrophil–Lymphocyte Ratio/Albumin Ratio for Death Within 21 Days

	AUC (95% CI)	Cut off	Sensitivity (%)	Specificity (%)	Accuracy (%)	Hosmer–Lemeshow test
PPI	0.71 (0.62–0.78)	6.0	59	68	62	*p* = 0.110
OPS	0.73 (0.63–0.78)	3.0	88	47	77	*p* = 0.311
NLR/Alb	0.72 (0.61–0.83)	0.18	72	73	71	*p* = 0.236

AUC, area under the curve; CI, confidence interval.

## Discussion

We reported the usefulness of PPI, OPS, and NLR/Alb, which correlate with the period until death and can predict death within 21 days in patients without cancer receiving home-visit palliative care.

In terminally ill cancer patients, when a PPI of 6.5 or higher was adopted as a cutoff point, death within 21 days was predicted with a sensitivity of 80% and a specificity of 85%. PPI applied to patients without cancer who received home-visit palliative care by changing the cutoff from 6.5 to 6.0, but the sensitivity and specificity in our study (AUC: 0.71; 95% CI: 0.62–0.79; sensitivity 59%; specificity 68%) were lower than that previously reported for cancer patients.^5^ With an OPS cutoff of 3.0, which is the same as that for cancer patients in a previous report,^6^ the sensitivity and specificity in our study were 88% and 47%, respectively.

An NLR/Alb cutoff of 0.1 was used in the first report but varied based on the report. In this study, when an NLR/Alb of 0.18 was adopted as a cutoff point, death within 21 days was predicted with an AUC of 0.72 (95% CI: 0.61–0.83), sensitivity of 72%, and specificity of 73%. No significant differences were found between the three indices using decision curve analysis (Fig. 3). These indicators should be used depending on the facility and patient situation, including whether regular blood tests are possible. Although paying attention to bias in sensitivity and specificity is necessary, they help support decision making regarding treatment recuperation and strategies over a short period.

Only Groups A and B by PPI displayed no significant differences in the analysis of survival curves for PPI, OPS, and NLR/Alb. The difference was not considered significant as the patient population had a high basal PPI (5.5 [3.5–6.5]), and the number of patients in Group A (PPI 0–2.0) was small (*n* = 6).

Prognosis prediction for patients without cancer is similar to that of patients with cancer; however, there are some difficulties. Specific prognostic indicators for patients without cancer have been explored for each disease and are not universal. The Body mass index,airflow Obstruction, Dyspnea, Exercise capacity (BODE) index^14^ for chronic obstructive pulmonary disease, Seattle Heart Failure Model (SHFM)^15^ and Meta-Analysis Global Group in Chronic Heart Failure (MAGGIC) risk score^16^ for chronic heart failure, and Child–Pugh score^17^ and Model for End-Stage Liver Disease (MELD) score^18^ for cirrhosis are useful for predicting disease-specific prognosis in clinical practice, but cannot be used for other diseases or generalized.

Few reports explore indicators that can be applied to all patients without cancer. Glare et al. reported that three groups of patients without cancer classified according to PaP score showed different survival curves, independent of diagnosis.^19^ Downar et al. reported that the question (“Would I be surprised if this patient died in specific time frame”) could predict death from 6 to 18 months (AUC: 0.77; 95% CI: 0.74–0.80), with a sensitivity of 60.7% (95% CI: 52.6–68.1) and specificity of 75.9% (95% CI: 67.6–82.6).^20^

In this study, we reported the usefulness of PPI, OPS, and NLR/Alb for all patients without cancer, including those with chronic respiratory disease, chronic heart failure, and chronic liver disease. Although the AUC was approximately the same, the sensitivity and specificity differ based on the cutoff, and the indicators should be used depending on the situation.

Previously reported indicators cannot be easily applied to home-visit palliative care. The BODE index requires a forced expiratory volume in one second as a percentage of forced vital capacity (FEV1%).^14^ SHFM and MAGGIC risk scores need echocardiographic findings.^15,16^ Moreover, the Advanced Dementia Prognostic Tool (ADEPT) score^21^ for patients with advanced dementia requires the evaluation of 12 variables. These indicators are complicated and unsuitable for rapid evaluation and tracking changes in home palliative care. PPI is evaluated solely based on physical findings and can be evaluated repeatedly and quickly.

The OPS requires blood tests, but consists of seven variables and can be easily evaluated. NLR/Alb is an objective indicator that can also be evaluated using blood tests. If blood tests are available, evaluating these indicators simultaneously may improve predictive capacity.

Existing indicators for predicting prognosis are mostly established on a monthly to yearly basis and cannot predict the daily to weekly prognoses for all patients without cancer. The OPTIMIZE-HF risk score,^22^ GWTG-HF risk score,^23^ and SOB-ASAP score^24^ are models for predicting the risk of short-term mortality among hospitalized patients with acute heart failure and are capable of predicting short-term prognosis. However, there have been no reports of short-term prognostic indicators for patients without cancer who are not hospitalized with acute heart failure.

This study has some limitations. As this was a retrospective study, the subjective variables of PPI and OPS were evaluated using medical records. The number of patients and evaluations per person was relatively small, and the total number of evaluations was only 182 times. Therefore, validation cohorts and prospective studies with larger number of patients are needed in the future. Each patient was evaluated multiple times in this study. Since repeated measure analysis was not used, intragroup variation caused by differences in conditions within the same individual was not taken into account. It should be incorporated into the analysis method in additional studies.

## Conclusions

PPI, OPS, and NLR/Alb were useful in predicting the survival period and short-term prognosis within 21 days for patients without cancer who received home-visit palliative care. The use of these indicators may provide decision-making support for patients receiving home-visit palliative care.

## Supplementary Material

Supplemental data

Supplemental data

Supplemental data

## Notes

**Cite this article as:** Hori T, Aihara K-I, Ishida K, Inaba K, Inaba K, Kaneko Y, Kawahito K, Bekku S, Hosoki M, Mori K, Itami K, Katsuse M, Hanaoka Y, Kageji T, Uraoka H, and Nakamura S (2024) Usefulness of palliative prognostic index, objective prognostic score, and neutrophil–lymphocyte ratio/albumin ratio as prognostic indicators for patients without cancer receiving home-visit palliative care: a pilot study at a community general hospital, *Palliative Medicine Reports* 4:1, 142–149, DOI: 10.1089/pmr.2023.0096.

## Abbreviations Used

ADEPT: Advanced Dementia Prognostic Tool
AIDS: acquired immunodeficiency syndrome
AUC: area under the curve
CI: confidence interval
ECOG PS: Eastern Cooperative Oncology Group Performance Status
LDH: lactate dehydrogenase
MAGGIC: Meta-Analysis Global Group in Chronic Heart Failure
MELD: Model for End-Stage Liver Disease
NLR/Alb: neutrophil–lymphocyte ratio/albumin ratio
OECD: Organization for Economic Cooperation and Development
OPS: objective prognostic score
PaP: palliative prognosis
PPI: palliative prognostic index
SD: standard deviation
SHFM: Seattle Heart Failure Model
WBC: white blood cell
