# Supplementary material for: Usefulness of Palliative Prognostic Index, Objective Prognostic Score, and Neutrophil–Lymphocyte Ratio/Albumin Ratio As Prognostic Indicators for Patients Without Cancer Receiving Home-Visit Palliative Care: A Pilot Study at a Community General Hospital
Source: Palliat Med Rep. 2024 Apr 4;5(1):142–9. doi: 10.1089/pmr.2023.0096 (PMC11002559; doi:10.1089/pmr.2023.0096)
Supplement: Supplemental data [file Suppl_TableS1.docx]

**Supplementary Table 1. Description of Palliative Prognostic Index (PPI)**

| Parameter | Assessment | Partial score |
| --- | --- | --- |
| Palliative Performance Scale | 10-20 | 4.0 |
|  | 30-50 | 2.5 |
|  | 60-100 | 0.0 |
| Oral intake | Mouthfuls or less | 2.5 |
|  | Reduced but more than mouthfuls | 1.0 |
|  | Normal | 0.0 |
| Edema | Present | 1.0 |
| Dyspnea at rest | Present | 3.5 |
| Delirium | Present | 4.0 |
