# Supplementary material for: Usefulness of Palliative Prognostic Index, Objective Prognostic Score, and Neutrophil–Lymphocyte Ratio/Albumin Ratio As Prognostic Indicators for Patients Without Cancer Receiving Home-Visit Palliative Care: A Pilot Study at a Community General Hospital
Source: Palliat Med Rep. 2024 Apr 4;5(1):142–9. doi: 10.1089/pmr.2023.0096 (PMC11002559; doi:10.1089/pmr.2023.0096)
Supplement: Supplemental data [file Suppl_TableS2.docx]

**Supplementary Table 2. Description of Palliative Performance Scale (PPS)**

| % | Ambulation | Activity level and disease symptom | Activities of daily living (ADL) | Oral intake | Level of consciousness |
| --- | --- | --- | --- | --- | --- |
|  |  |  |  |  |  |
| 100 | Full | Normal activity and no symptoms | Full | Normal | Normal |
|  |  |  |  |  |  |
| 90 |  | Normal activity with some symptoms |  |  |  |
|  |  |  |  |  |  |
| 80 |  | Able to do normal activity with effort |  | Normal or reduced |  |
|  |  |  |  |  |  |
| 70 | Reduced | Unable to do normal job or work due to some symptoms |  |  |  |
|  |  |  |  |  |  |
| 60 |  | Unable to do hobby or housework due to obvious symptoms | Occasional assistance needed |  | Normal or confusion |
|  |  |  |  |  |  |
| 50 | Mainly sit or lie | Unable to do any work due to severe symptoms | Considerable assistance needed |  |  |
|  |  |  |  |  |  |
| 40 | Mainly in bed |  | Mainly assistance |  | Normal or confusion or drowsy |
|  |  |  |  |  |  |
| 30 | Bed bound |  | Total care | Reduced |  |
|  |  |  |  |  |  |
| 20 |  |  |  | Minimal |  |
|  |  |  |  |  |  |
| 10 |  |  |  | Mouth care only | Drowsy or coma |
|  |  |  |  |  |  |
| 0 | Death | | | | |
|  |  |  |  |  |  |
