# Supplementary material for: Usefulness of Palliative Prognostic Index, Objective Prognostic Score, and Neutrophil–Lymphocyte Ratio/Albumin Ratio As Prognostic Indicators for Patients Without Cancer Receiving Home-Visit Palliative Care: A Pilot Study at a Community General Hospital
Source: Palliat Med Rep. 2024 Apr 4;5(1):142–9. doi: 10.1089/pmr.2023.0096 (PMC11002559; doi:10.1089/pmr.2023.0096)
Supplement: Supplemental data [file Suppl_TableS3.docx]

**Supplementary Table 3. Description of Objective Prognostic Score (OPS)**

| Parameter | Assessment | Partial score |
| --- | --- | --- |
| ECOG PS | 4 | 1.0 |
|  | 1-3 | 0.0 |
| Anorexia | Present | 1.0 |
| Dyspnea at rest | Present | 1.0 |
| WBC | ＞11,000/μL | 1.0 |
| Total Bil | ＞2.0mg/dL | 1.0 |
| Cre | ≥1.5mg/dL | 2.0 |
| LDH | ≥502 IU/L | 1.0 |
| Anorexia was defined as taking less than five spoonfuls per meal or less than one-third of a routine meal. | | |

Abbreviations: ECOG PS: Eastern Cooperative Oncology Group Performance Status; WBC: white blood cells; Bil: bilirubin; Cre: creatinine; LDH: lactate dehydrogenase
